# Acupuncture Promotes Angiogenesis after Myocardial Ischemia through H3K9 Acetylation Regulation at VEGF Gene

**DOI:** 10.1371/journal.pone.0094604

**Published:** 2014-04-10

**Authors:** Shu-Ping Fu, Su-Yun He, Bin Xu, Chen-Jun Hu, Sheng-Feng Lu, Wei-Xing Shen, Yan Huang, Hao Hong, Qian Li, Ning Wang, Xuan-Liang Liu, Fanrong Liang, Bing-Mei Zhu

**Affiliations:** 1 Key Laboratory of Acupuncture and Medicine Research of Ministry of Education, Nanjing University of Chinese Medicine, Nanjing, Jiangsu, China; 2 School of Acupuncture and Tuina, Chengdu University of Traditional Chinese Medicine, Chengdu, Sichuan, China; 3 School of Information Technology, Nanjing University of Chinese Medicine, Nanjing, Jiangsu, China; Indiana University School of Medicine, United States of America

## Abstract

**Background:**

Acupuncture exerts cardioprotective effects on several types of cardiac injuries, especially myocardial ischemia (MI), but the mechanisms have not yet been well elucidated. Angiogenesis mediated by VEGF gene expression and its modification through histone acetylation has been considered a target in treating myocardial ischemia. This study aims to exam whether modulation of angiogenesis through H3K9 acetylation regulation at VEGF gene is one possible cardioprotective mechanism of acupuncture.

**Results:**

We generated rat MI models by ligating the left anterior descending coronary artery and applied electroacupuncture (EA) treatment at the Neiguan (PC6) acupoint. Our results showed that acupuncture reversed the S-T segment change, reduced Q-wave area, decreased CK, CK-MB, LDH levels, mitigated myocardial remodeling, and promoted microvessel formation in the MI heart. RNA-seq analysis showed that VEGF-induced angiogenesis signaling was involved in the modulation of EA. Western blot results verified that the protein expressions of VEGF, Ras, phospho-p44/42 MAPK, phospho-p38 MAPK, phospho-SAPK/JNK and Akt, were all elevated significantly by EA treatment in the MI heart. Furthermore, increased H3K9 acetylation was also observed according with the VEGF. ChIP assay confirmed that EA treatment could notably stimulate the recruitment of H3K9ace at the VEGF promoter.

**Conclusions:**

Our study demonstrates for the first time that acupuncture can effectively up-regulate VEGF expression through H3K9 acetylation modification directly at the VEGF promoter and hence activate VEGF-induced angiogenesis in rat MI models. We employed high throughput sequencing in this study and, for the first time, generated genome-wide gene expression profiles both in the rat MI model and in acupuncture treatment.

## Background

Acupuncture, the most well-known complementary and alternative medical approach, has been applied to prevent and cure diseases, including angina[1], palpitation [2], stroke [3], and dysfunction of the left cardiac function in coronary heart disease (CHD) [4] for more than 3000 years. Past records indicate that needling at Neiguan (PC6) acupoint exerts cardioprotective effects on several kinds of ischemic heart injuries, including cardiac surgery, myocardial ischemia-reperfusion, and acute myocardial ischemia by improving hemodynamics, microcirculation and energy metabolism, as well as decreasing susceptibility to ventricular tachycardia [5]–[13].

Angiogenesis is the generation and expansion of blood vessels from a preexisting vascular network under endogenous or exogenous stimuli and can be used as a therapeutic target for myocardial ischemia. Myocardial function relies on sufficient blood supply and the normal capillary/myocardial fiber ratio of 1∶1 (one capillary to one myocardial fiber) in an adult heart. In the case of hypoxia, angiogenesis is triggered by insufficient blood supply [14], [15]. Vascular endothelial growth factor (VEGF), one of angiogenic factors, is involved in all phases of angiogenesis, including generation of angiogenic vascular sprout, stabilization of immature neovascular sprout, and maturation of physiological angiogenesis [14]. In ischemic areas, VEGF expression can increase up to 30 times in a few minutes by the accumulation of hypoxia-inducible factor-1 (HIF-1), which activates VEGF transcription by binding to the hypoxia response element (HRE) on the VEGF promoter [16]. However, in the absence of VEGF, the emerging neovascular sprout is destabilized and eventually regresses due to the lack of VEGF signals [17]. All above evidence indicates that VEGF plays an important role in stimulating both physiological and pathological angiogenesis.

In order to investigate whether acupuncture at the PC6 acupoint can regulate VEGF expression and angiogenesis, as well as the mechanism of gene regulation by acupuncture, we generate rat MI models by ligating the left anterior descending coronary artery and applying electroacupuncture (EA) treatment at the PC6 acupoint. In the present study, we demonstrated for the first time that acupuncture can effectively up-regulate VEGF expression through H3K9 acetylation modification directly at the VEGF promoter and hence contribute to angiogenesis in the rat MI model. Our results suggest that post-translation histone modification plays a role in the cardioprotective effects of EA following MI injury.

## Materials and Methods

### Antibodies and reagents

Antibodies for VEGF, alpha-smooth muscle actin (α-SMA), and Histone H3 were purchased from Abcam (Cambridge, UK). Antibody for CD34 was obtained from Santa Cruz Biotechnology (Santa Cruz, CA, USA). Antibodies for Acetyl-Histone H3 (Lys9) (H3K9ace), Ras, Akt GAPDH and Phospho-MAPK family antibody sampler kit, were obtained from Cell Signaling. Dynabeads protein A was obtained from Invitrogen. Supersignal west pico chemiluminescent substrate was purchased from Pierce (Rockford, IL). Truseq RNA sample prep kit-v2, Truseq DNA sample prep kit-PCR box, c-BOT Multiplex re-hybridization plate and Truseq Sbs kit V3 were all purchased from Illumina.

### Animals and grouping

Adult male Sprague Dawley (SD) rats (250–300 g), supplied by the Experimental Animal Center of Nanjing University of Chinese Medicine, were used for this study. All rats were fasted (with free access to water) for 24 h before surgical operation and randomized into control, control+EA (CEA), MI and EA groups. The study was approved by the Institutional Animal Care and Use Committee of Nanjing University of Chinese Medicine, and all procedures were conducted in accordance with the guidelines of the National Institutes of Health Animal Care and Use Committee.

### *In vivo* MI

The rat MI model was created after thoracotomy by applying permanent ligation of the left anterior descending coronary artery (LAD) as described previously [18]. Rats were anaesthetized by inhalation of 5% isoflurane and maintained with 2% isoflurane in a mixture of 70% N2O and 30% O2, then subjected to a tracheal cannula, carotid artery intubation, and thoracotomy between the 3rd and 4th intercostal spaces on the left side of the chest along the sternum, and permanent LAD ligation. In the control group, the same procedure was performed except for the LAD ligation. Blood pressure and ECG were monitored throughout the operation.

### Electrocardiogram (ECG) recording of electrode resettlement

To evaluate situation of myocardial ischemia we monitored chest lead of rats. ECG was continuously monitored before, during, and after myocardial ischemia by the use of a computerized PowerLab system (ADInstruments, Australia). For recording ECG of the anterior precordial lead V3–V4, rats were anaesthetized by inhalation of isoflurane as mentioned above, two stainless steel electrodes were placed beneath the skin close and to the left of the xiphoid-process and the dorsal neck, respectively. The reference electrode was placed beneath the skin of the right hindlimb. The areas of Q-wave in ECG [width(s) × voltage (mv)] was recruited as an index of ischemia, and were compared statistically in each group on day 1, day 2, and day 7 after surgery. In addition, the S-T segment of ECG elevated over 100 µV from the baseline was recruited as an index of ischemia. △S-T value, the absolute value of S-T segment above and below baseline of each group before surgery and 7 days after surgery, were compared statistically.

### EA intervention

As previously described, bilateral PC6 which were located at a point 1.5 cm proximal to the palm crease just above the median nerve [12]. Two acupuncture needles (Gauge-28, 0.5 cm) were separately inserted into each acupoint located on each upper limb, and an electrical current was provided to the needles through an electrical stimulator with a stimulus isolation unit (Han Acuten, WQ1002F, Beijing, China) with parameters of 2/15 Hz at an intensity level of 1 mA, for a total stimulation period of 20 minutes (Fig. S5). The acupuncture needle, 15 mm long and 0.3 mm in diameter, was inserted 2–3 mm into the subcutis. Rats in EA and CEA groups were put under anesthesia after MI or sham operation and were applied EA treatment once a day, for a total of 7 days. Rats were then euthanized with intravenous injections of high-dose pentobarbitone, and cardiac tissues were harvested for morphological or molecular analyses.

### Myocardial enzyme analysis

Five milliliters of blood was collected from the jugular vein with a procoagulant on day 1, day 2, and day 7 after surgery and centrifuged (10,000 g, 10 minutes, 4°C). Serum was extracted and analyzed for creatine kinase (CK), creatine kinase-MB (CK-MB), and lactate dehydrogenase (LDH) levels with a biochemistry analyzer.

### Histological analysis and Immunofluorescence staining

After sacrificing the rats via intravenous injection of high-dose pentobarbitone at the end of the 7-day EA treatment, cardiac tissues and sections were prepared from the paraffin-embedded hearts. The sections from the apex, mid-left ventricle (LV), and base were stained with H&E and Masson's trichrome according to the manufacturer's protocols (Sigma). Images were acquired using a light microscope (Nikon, Japan). The area of infarction and the total area of LV were traced manually and measured using Image J software (National Institutes of Health Bethesda, MD). Scar size was then measured according to the reference [19].

To assess the angiogenesis, sections of the heart were used for CD34, α-SMA and VEGF immunohistochemistry. Circulating endothelial progenitor cells (cEPCs) were identified by CD34 antibody, smooth muscle cells were identified by α-SMA, and endothelial cells were identified by VEGF. Tissue sections were placed at 70°C for 30 min and subsequently immersed in xylene and ethanol at decreasing concentrations. Slides were then washed in distilled water. After one hour of blocking with the buffer solution, sections were incubated overnight at 4°C with CD34 (1: 200, Abcam), α-SMA (1∶500, Abcam), or VEGF (1: 200, Abcam). After rinsing in PBS, slides were then incubated with the appropriate Alexa 568 Goat anti mouse secondary antibody (1∶400, Invitrogen) or Alexa 488 Goat anti rabbit secondary antibody (1∶400, Invitrogen) for two hours at room temperature; DAPI was then used to stain the nucleus. Sections were mounted with anti-fade fluorescence mounting medium (Dako, Denmark). Images were acquired using a fluorescent microscope at a constant exposure. Approximately 20 randomized fields of each tissue sections, taken at the mid plane of each heart, and containing infarct and border regions, were counted for the stained number of cells. The total numbers of VEGF^+^、 CD34^+^、 α-SMA^+^ cells from each group were calculated and normalized to the number of DAPI^+^ cells with Image J software.

### RNA-Seq and computational analysis for RNA-seq data

At the end of the 7-day EA treatment, total RNA was extracted by Trizol reagent (Invitrogen) from the harvested hearts. For RNA-Seq, RNA samples were prepared according to the TruSeq RNA Sample Preparation v2 protocol, and the DNA libraries were applied to the cluster generation and sequencing using c-BOT Multiplex re-hybridization plate and Truseq Sbs kit V3. Sequencing was performed using Illumina Hiseq 2000 (Illumina, USA).

After sequencing with HiSeq 2000 (Illumina), raw fastq files were extracted from Illumina BCL using the Illumina CASAVA program. The single-end reads of biological triplicates obtained from each sample were aligned to the rat reference genome (UCSC rn4 assembly) using the TopHat program [20], [21]. The Cufflinks program was used to assemble individual transcripts from RNA-seq reads that have been aligned to the genome and to qualify the expression level of each transcript. Differential transcripts expression analysis was performed with the Cuffdiff program. The gene's functional annotation and pathway were analyzed using the DAVID Bioinformatics Resources [22]. The raw data have been deposited onto NCBI'S Read Archhive (SRA) database and the accession number is GSE54132.

### Western blot

The cardiac tissues were obtained 7 days post-surgery and lyses with RIPA buffer supplemented with a protease inhibitor cocktail. Protein concentrations were determined using the BCA protein assay (Pierce). Equivalent amounts of protein (30 µg/lane) were resolved electrophoretically by SDS-polyacrylamide gels (10%) and transferred onto PVDF membranes. Nonspecific reactivity was blocked in 5% BSA in TBST (10 mM Tris-HCl, pH 7.5, 150 mM NaCl, 1% Tween-20) for one hour. Membranes were incubated with anti-VEGF (1∶1000), anti-H3K9ace (1∶1000), anti-Histone H3 (1∶1000), Akt(1∶1000), Ras(1∶1000), Phospho-p44/42 MAPK (1∶1000), Phospho-p38 MAPK (1∶1000), Phospho-SAPK-JNK(1∶1000), and GAPDH (1∶3000) overnight at 4°C, followed by incubation with secondary antibodies for one hour at room temperature. Proteins were visualized by using the supersignal west pico chemiluminescent substrate (Pierce).

### Chromatin immunoprecipitation (ChIP) assay

To determine histone modifications in the VEGF promoter region, chromatin immunoprecipitation (ChIP) assay was carried out [23]. In brief, the protein-chromatin lysate was fragmented to a length between 200 and 1,000 bp by sonication (Diagenode). The chromatin was precleaned by incubating with Dynabeads protein A and immunoprecipitated with rotation; at 4°C overnight with the antibody against anti-H3K9ace (1∶50). The precipitated chromatin was then harvested by Dynabeads protein A and eluted in buffer after a series of washes. The ChIP DNA was eluted and purified by phenol/chloroform method, to reach a final volume of 50 µl. 2 µL of the ChIP DNA was subjected to quantitative analysis by real-time PCR (qPCR). Primers were designed to amplify the promoter region between 500 bp upstream and 1000 bp downstream of TTS at the VEGF gene. The primer sequences are as follows: rat VEGF -485 to -317: forward 5′- CGTAACTTGGGCGAGCCG -3′: and reverse 5′- CGTAACTTGGGCGAGCCG -3′

rat VEGF −403 to −124: forward 5′-GTGTGTCTGGGTATAGTGTG-3′ and reverse 5′-GCCACTACTGCGAAATAGAAA-3′

rat VEGF -68 to +126: forward 5′- GTTTCCACAGGTCGTCTC -3′ and reverse 5′- GGGGAGTATGCTTATCTG -3′

rat VEGF +666 to +928: forward 5′-TGAGTCAAGAGGACAGAGAG-3′and reverse 5′-ATTACCAGGCCTCTTCTTCC-3′

Fold enrichment in the binding of H3K9ace to VEGF promoter regions in each immunoprecipitation was normalized to the indicated histone binding level in its corresponding input DNA. Two independent ChIP experiments were performed for each analysis, and qPCR was performed twice for each of the ChIP DNA sample. The standard deviation among the experiments did not exceed 10% of the average values. The average values were graphed to demonstrate the relative percent of occupancy.

### Statistical analysis

Data were presented as means ± standard deviation (SD). Statistics analysis was performed using SPSS 18.0; multiple group comparisons were made by ANOVA and two group comparison was determined using unpaired 2-tailed Student's t test. P<0.05 was considered statistically significant.

## Results

### EA at PC6 protected rat heart from MI injury

**Figure 1:**
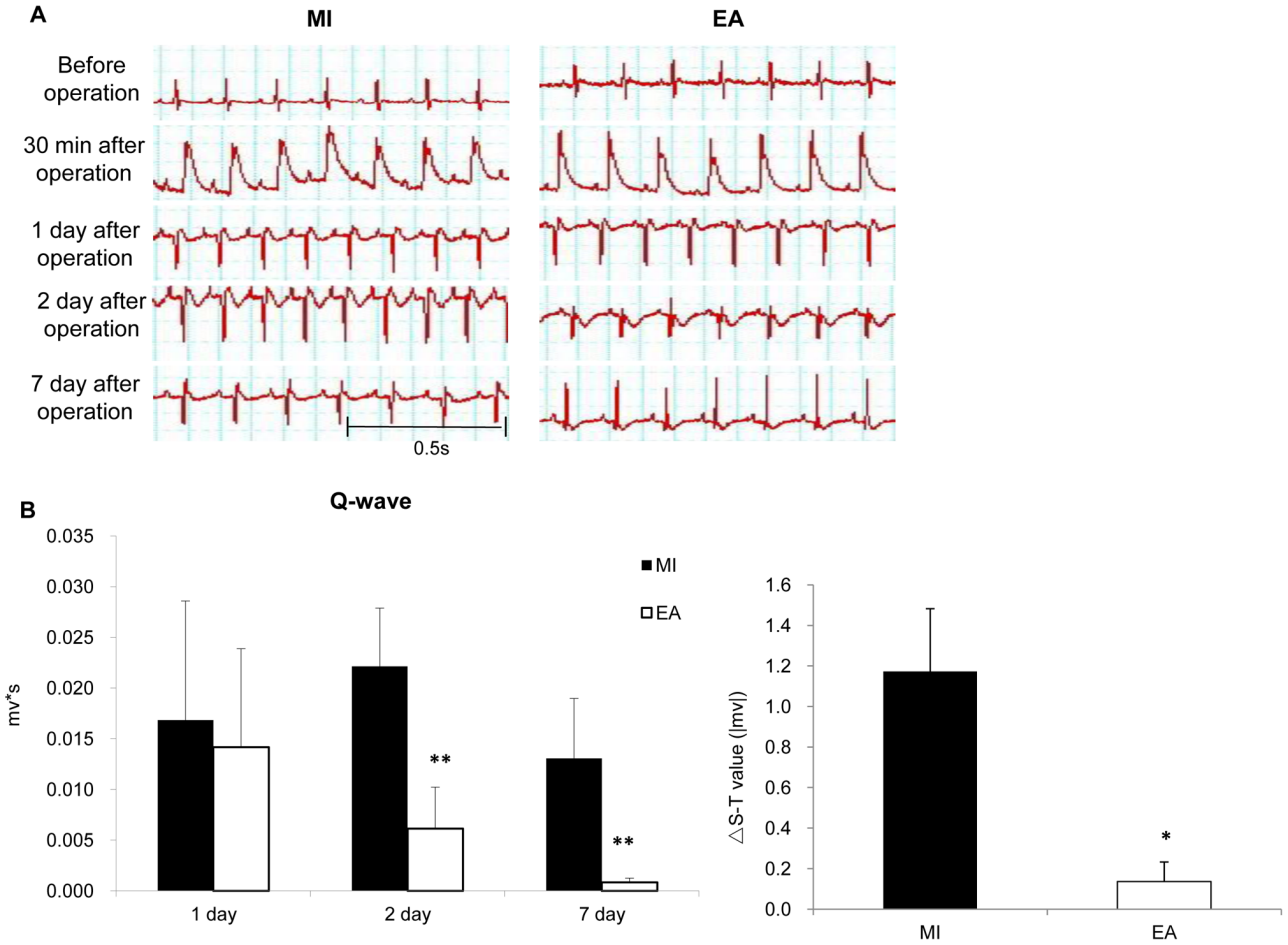
Effects of EA treatment on ECG recording of MI heart. ECG was continuously monitored before, during and after myocardial ischemia. A. Representative ECG recording in anterior precordial lead V3-V4. B. Quantification of Q-wave area value and △S-T value in each group. Data were expressed as means ± SD, n = 10,^ **^ *P*<0.01*vs* MI.

**Figure 2:**
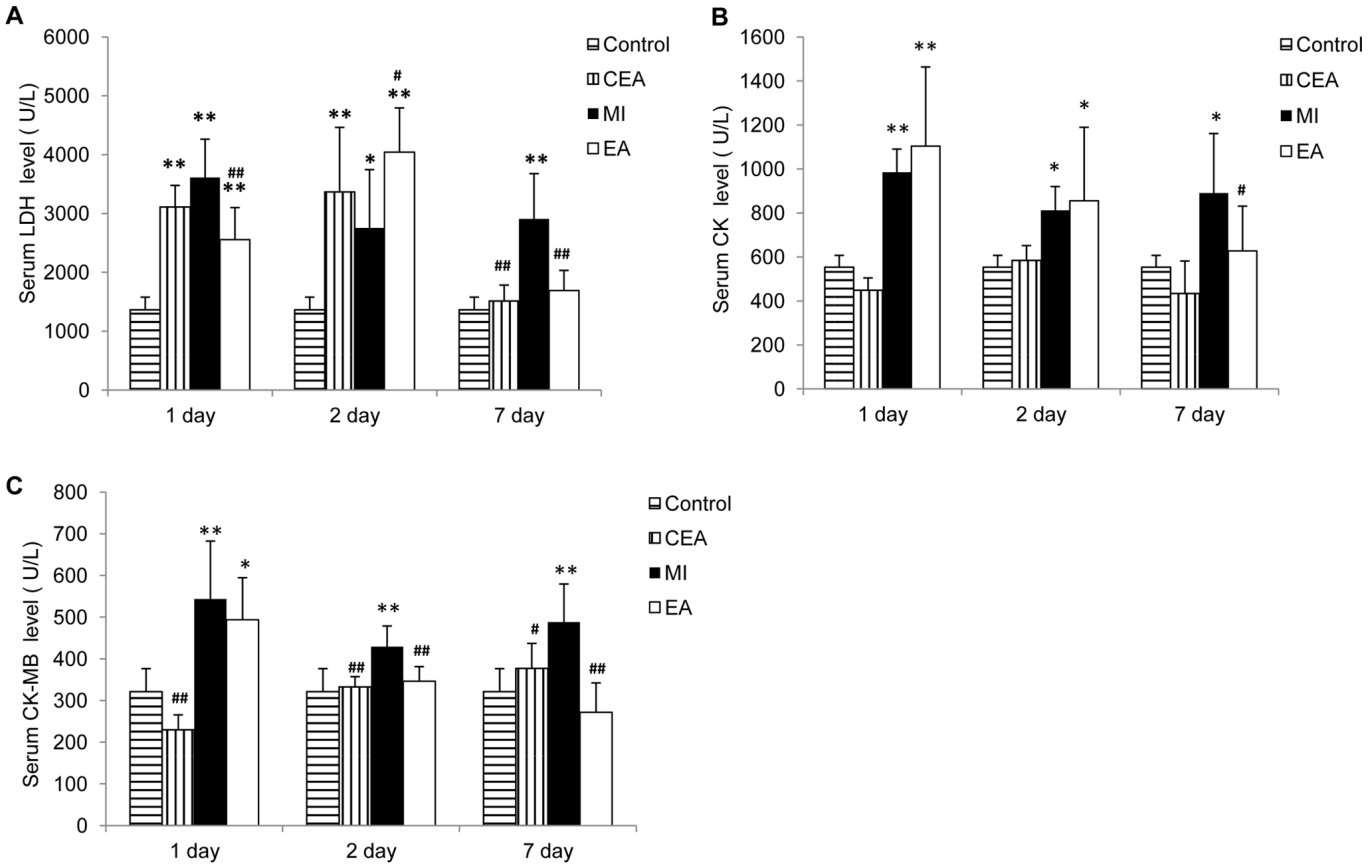
Effects of EA treatment on myocardial enzymes release of MI hearts. Serum was extracts and analyzed for LDH (A), CK (B) and CK-MB (C) levels by biochemistry analyzer on the 1^st^, 2^nd^ and 7^th^ day after operation. Data were expressed as means ± SD, n = 10, ^**^P<0.01, *P<0.05 vs. Control, ^##^ P<0.01, ^#^ P<0.01vs MI.

**Figure 3:**
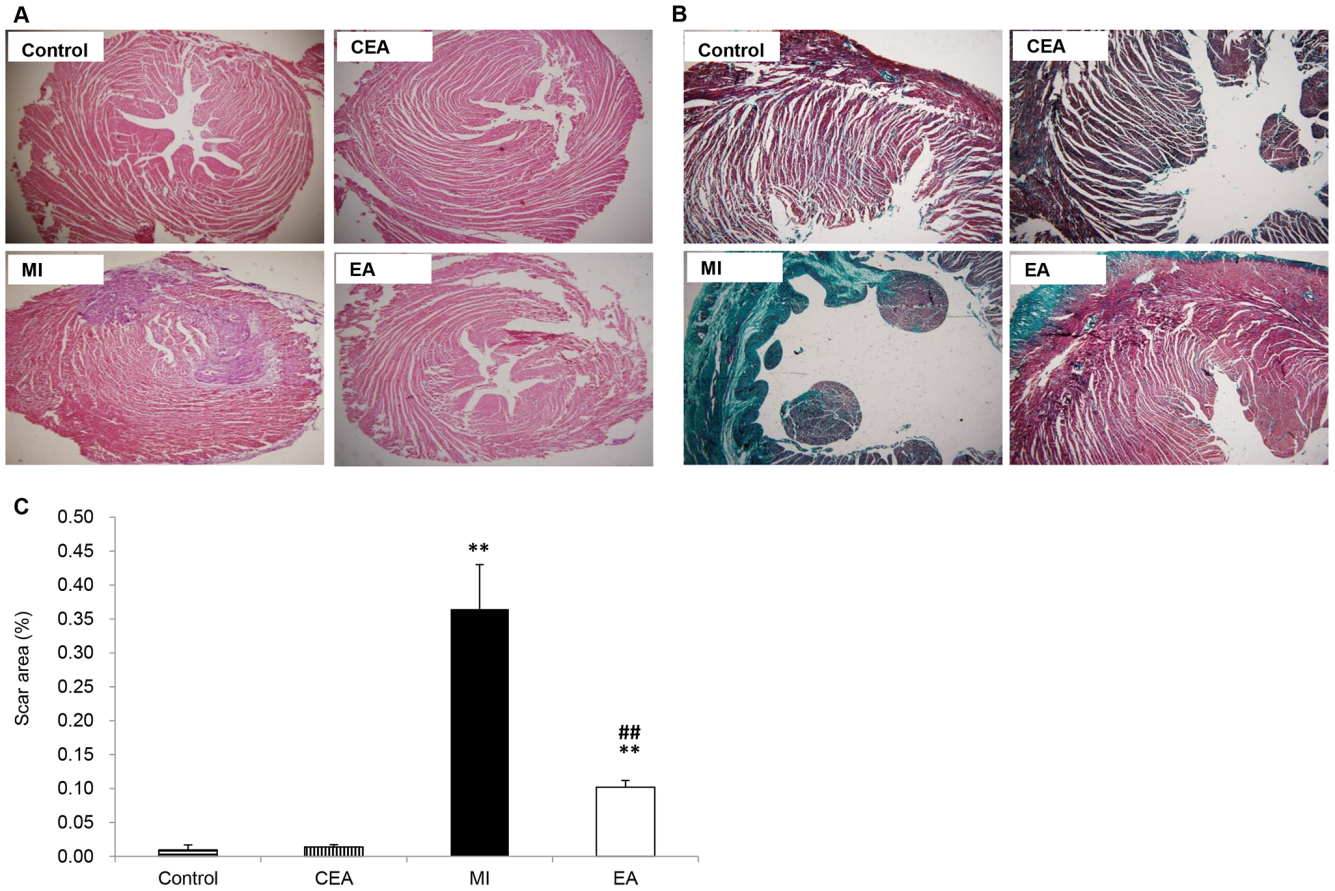
Evaluation of myocardial remodeling. A. Representative image of hematoxylin and eosin (H&E) staining of each group. B. Representative image of Masson's trichrome staining of each group, scale bar, 100 µm. C. Relative quantification of scar size. Data were showed as means ± SD, n = 5, ^*^P<0.01 vs. Control, ^#^ P<0.01vs MI.

To investigate protective effects of EA on ischemic injury induced by MI, we first observed the ECG's Q wave area, ST segment, myocardial damage, and myocardial enzyme levels. Our results showed that the diastolic and systolic blood pressure were both decreased after ligation (Fig.S1), the ST segment was noticeably elevated in both MI and EA groups in 30 min, and the representative necrotic Q-waves were appeared 1 day after operation, indicating the success of the MI model (Fig.1A). Q waves on the 2^nd^ day after operation was deeper than on the 1^st^ day, and was relieved on the 7^th^ day in MI group. However in the EA group, Q-wave areas were significantly decreased the on 2^nd^ and 7^th^ day after operation compared with the MI group. At the same time, we observed that the △S-T values were also significantly decreased in the EA group compared with the MI group (*P*<0.01) (Fig.1A and B). The levels of enzymes, which reflect acute myocardial injury, were measured from peripheral blood serum of the rats. Concentrations of LDH, CK, and CK-MB significantly increased in the MI model group from the 1^st^ day through the 7 days after operation. In the EA group, the enzymes concentrations decreased significantly to the level of the control group on the 7^th^ day after operation, and MI induced elevation of CK-MB was alleviated by EA at PC6 early on the 2^nd^ day after operation (Fig.2A, 2B, 2C). Additionally, EA applied on the control rats did not affect most of the enzymes except LDH, which was increased on the 1^st^ and 2^nd^ day after operation and went back to the normal level on 7^th^ day. Meanwhile, rat survival rate in the EA group was also improved compared with the MI model group (Fig.S2). Moreover, the extent of myocardial damage and remodeling was assessed by H&E and Masson's trichrome staining (Fig.3A, 3B, S3 and S4). In the control group and CEA group, myocardial cells and fibers showed integrate structure without inflammatory cell infiltration and swelling. The ischemic heart in the MI group displayed myocardial fiber fracture, edema, and degeneration, as well as a small amount of necrosis, infiltration of inflammatory cells, and fibrosis. EA significantly reduced cardiac tissue damage compared with the MI group; myocardial cell structure was reserved with decreased edema and degeneration of myocardial fibers. Statistical analysis showed that the infarct scar area was decreased from 36.46% to 10.21% in the EA group compared to the MI group (*P*<0.001) (Fig.3C).

### Genome-wide profiling of gene expression under EA treatment on MI heart in rats

**Figure 4:**
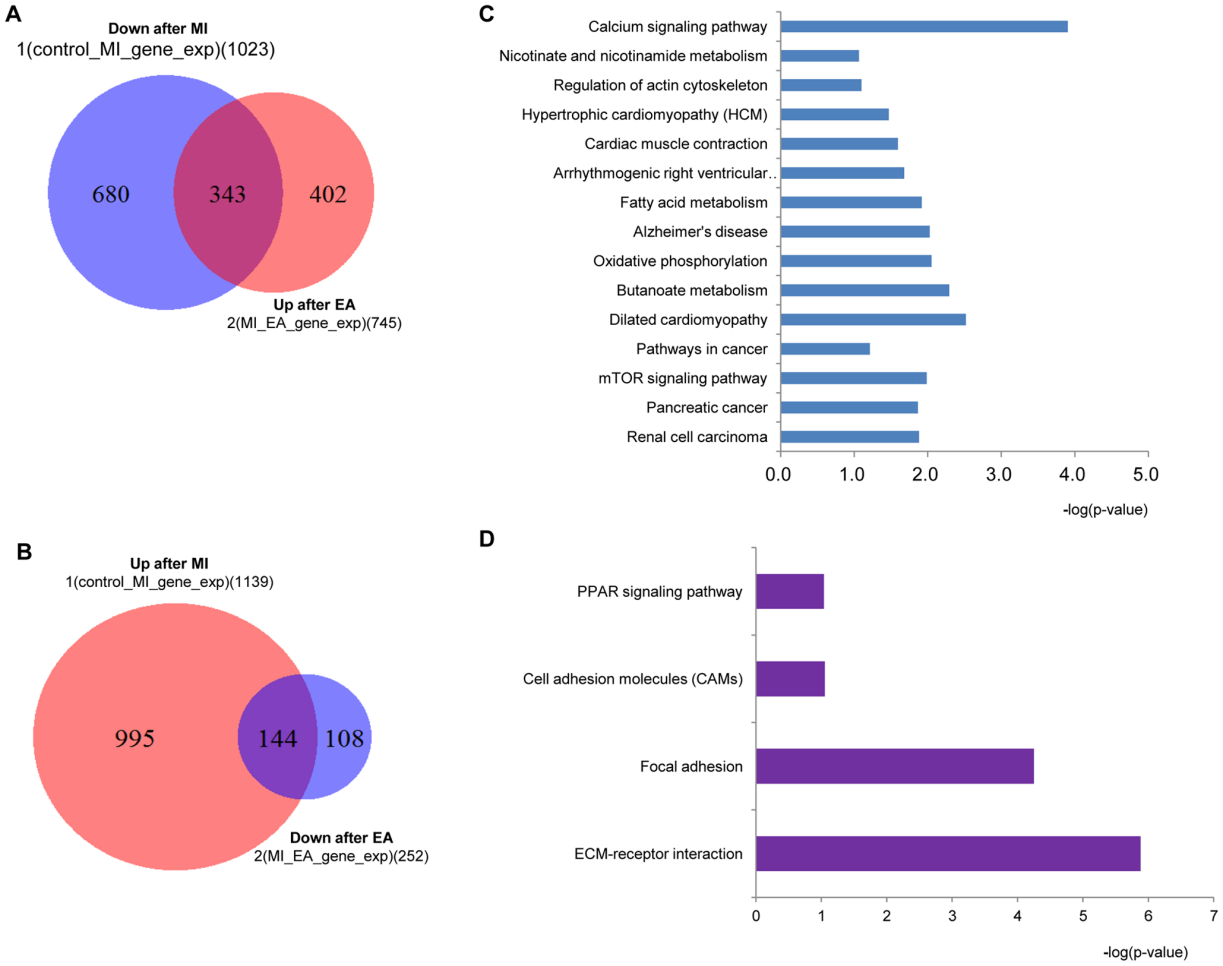
Genome-wide association study results. At the end of EA treatment for 7 days, the total RNA was extracted from the harvested hearts for RNA-Seq analysis. A and B, Venn diagrams of regulated gene numbers by MI and EA treatment. A. Overlapped area represents increased gene number (343) in EA group but decreased in MI group; B. Overlapped area represents decreased gene number (144) in EA group but increased in MI group. C. The top 15 pathways of these genes involved in the 343 genes in A; D. Major pathways of the 144 genes shown in B.

After we confirmed the cardioprotective effect of EA on MI injury, we further investigated its possible mechanisms. RNA-seq by next generation sequencing was used to identify the rat genome-wide alterations after MI and EA treatment (The raw data shown in GEO with the number GSE54132). Following MI, we saw at least a two-fold increase in the expression of 1023 genes and a similar decrease in the expression of 1139 genes. Interestingly, EA treatment reversed some of the gene expression changes induced by MI; 343 out of 1023 genes whose expressions decreased in the MI group were increased, and 144 out of 1139 genes whose expressions increased in the MI group were decreased post-EA treatment. In addition to these co-regulated genes, other gene expression levels that were not influenced by MI were also regulated by EA (Fig 4A and 4B). Pathway analysis indicated that these co-regulated genes were mainly involved in the calcium signaling pathway, cardiomyopathy related pathways, and cancer related pathways (Fig 4C and 4D), such as VEGF, an angiogenic factor, and VEGF-activated signaling component, including Ras, Akt and MAPK family members, decreased in the MI group, but significantly increased in the EA group (Table 1).

**Table 1:** VEGF signaling related gene expressions (FPKM).

gene	Control	MI	EA	MI/Control	EA/MI
Vegfb	92.28	52.72	154.27	0.57	2.93
Vegfa	26.28	12.61	26.49	0.48	2.10
Rassf3	3.59	1.40	58.19	0.39	16.22
Map3k13	4.43	1.92	6.78	0.43	3.52
Akt2	70.30	35.712	63.50	0.51	1.78
Aktip	17.43	9.47	18.00	0.54	1.90
Mapk1	25.24	11.01	24.03	0.44	2.18
Mapkapk2	75.60	51.93	155.46	0.69	3.00
Mapkbp1	0.76	0.58	1.20	0.77	2.06
Mapk3	74.65	58.19	81.67	0.78	1.40

### EA at PC6 promoted angiogenesis in ischemic myocardium through activation of VEGF signaling

**Figure 5:**
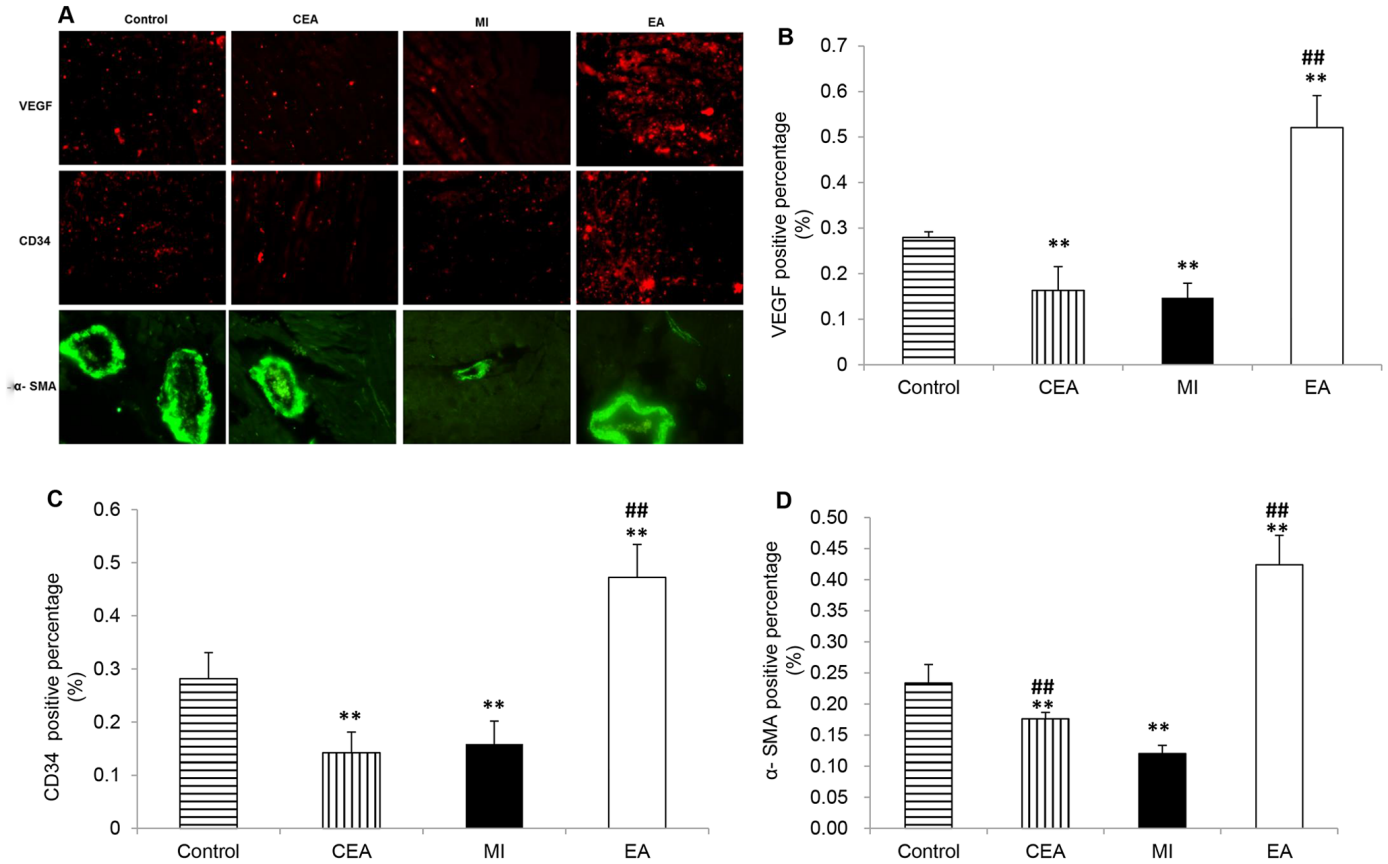
Effect of EA treatment on angiogenesis in MI hearts. A. Representative immunofluorescence staining images of VEGF (red), CD34 (red) and α-SMA (green) in each group, scale bars, 100 µm. B to D, Quantitative analysis of VEGF-positive, CD34-positive and α-SMA–positive percentages. Data were shown as means ± SD, n = 5, ^**^P<0.01 vs. Control, ^##^ P<0.01vs MI.

**Figure 6:**
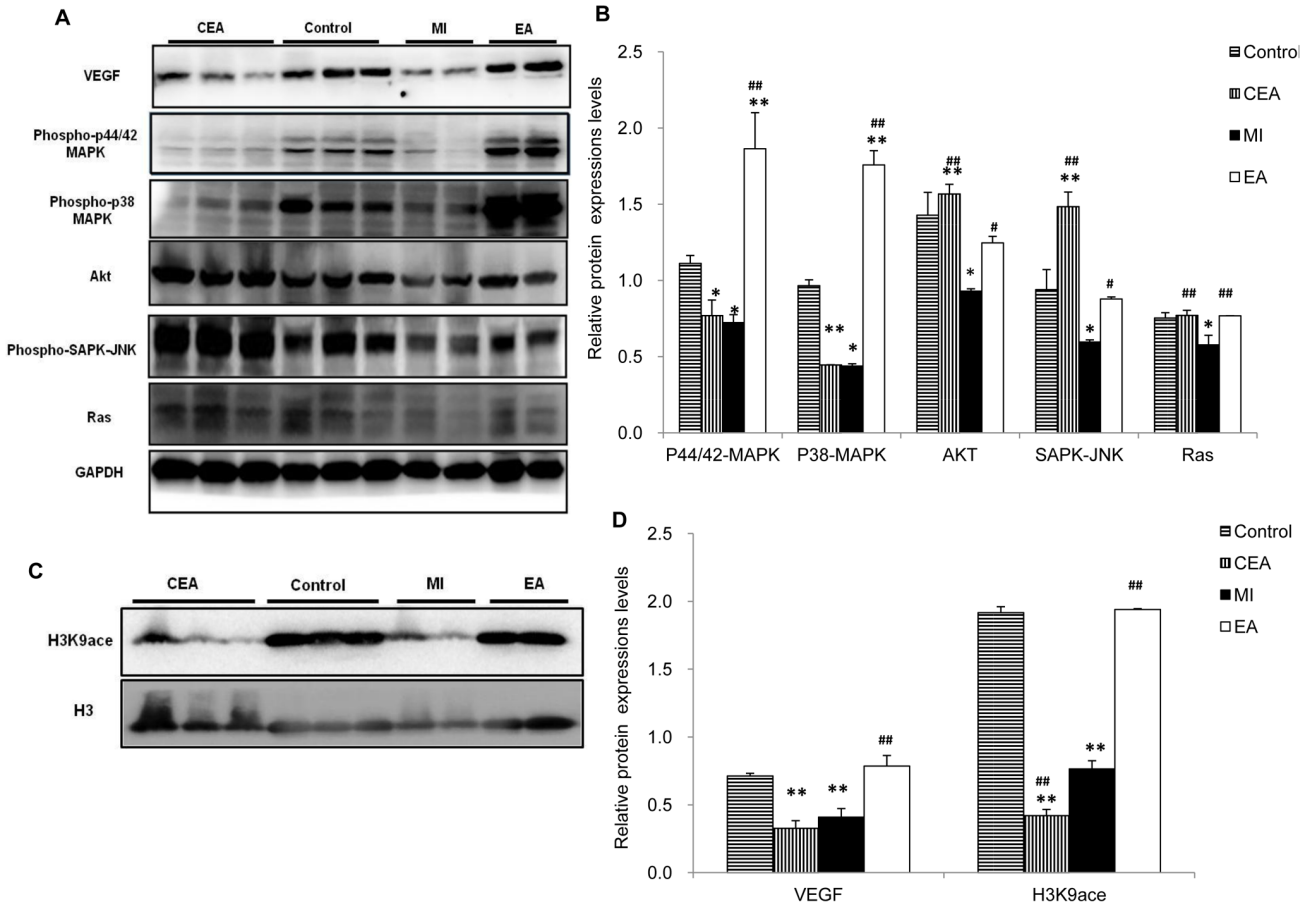
Detection of protein expressions related to VEGF-induced angiogenesis and H3K9 acetylation level. A and C. Representative western blot results of VEGF signaling proteins and H3K9ace in each group. B and D. Quantitative analysis of VEGF signaling proteins and H3K9 ace. Data were showed as means ± SD (n = 4∼6), ^*^P<0.05, ^**^P<0.01 vs. Control, ^#^ P<0.05, ^#^^#^ P<0.01 vs. MI.

To verify that angiogenesis is a possible mechanism in the EA-induced myocardioprotective effect; angiogenic responses were examined by immunofluorescence staining of CD34, α-SMA and VEGF. CD34-, α-SMA- and VEGF- positive cells were presented to the circulating endothelial progenitor cells (cEPCs), vascular smooth muscle cells, and endothelial cells, respectively. As shown in Fig.5A and 5C, EA treatment caused a marked increase in CD34-positive cEPCs, suggesting increased vessel density in EA-treated hearts compared with the MI group (*P*<0.01). In the border and center regions of the infarct zone, α-SMA positive vascular density staining was observed, indicating that EA treatment significantly increased α-SMA positive ratio compared with the MI group (Fig.5A and 5D). Likewise, we also observed a significant increase in VEGF-positive staining after treatment with EA, whereas in the center and border regions of the MI heart VEGF protein level markedly decreased (*P*<0.01) (Fig.5A and 5B), suggesting that VEGF-induced angiogenesis is involved in the EA treatment. Western blot results showed that VEGF protein expression was decreased obviously, accompanying with the decrease of Ras, phospho-p44/42 MAPK, phospho-p38 MAPK, phospho-SAPK/JNK and Akt proteins in the MI group. In contrast, EA treatment inhibited MI induced decrease of these proteins; the phospho-p44/42 MAPK and phospho-p38 MAPK expression were even overexpressed compared with the control group. Surprisingly, in CEA group, the expressions of VEGF, phospho-p44/42 MAPK and phospho-p38 MAPK were also decreased, meanwhile phospho-SAPK/JNK and Akt expressions were significantly increased. (Fig.6A, 6B, 6D)

### Elevation of VEGF are regulated directly by H3K9 acetylation

**Figure 7:**
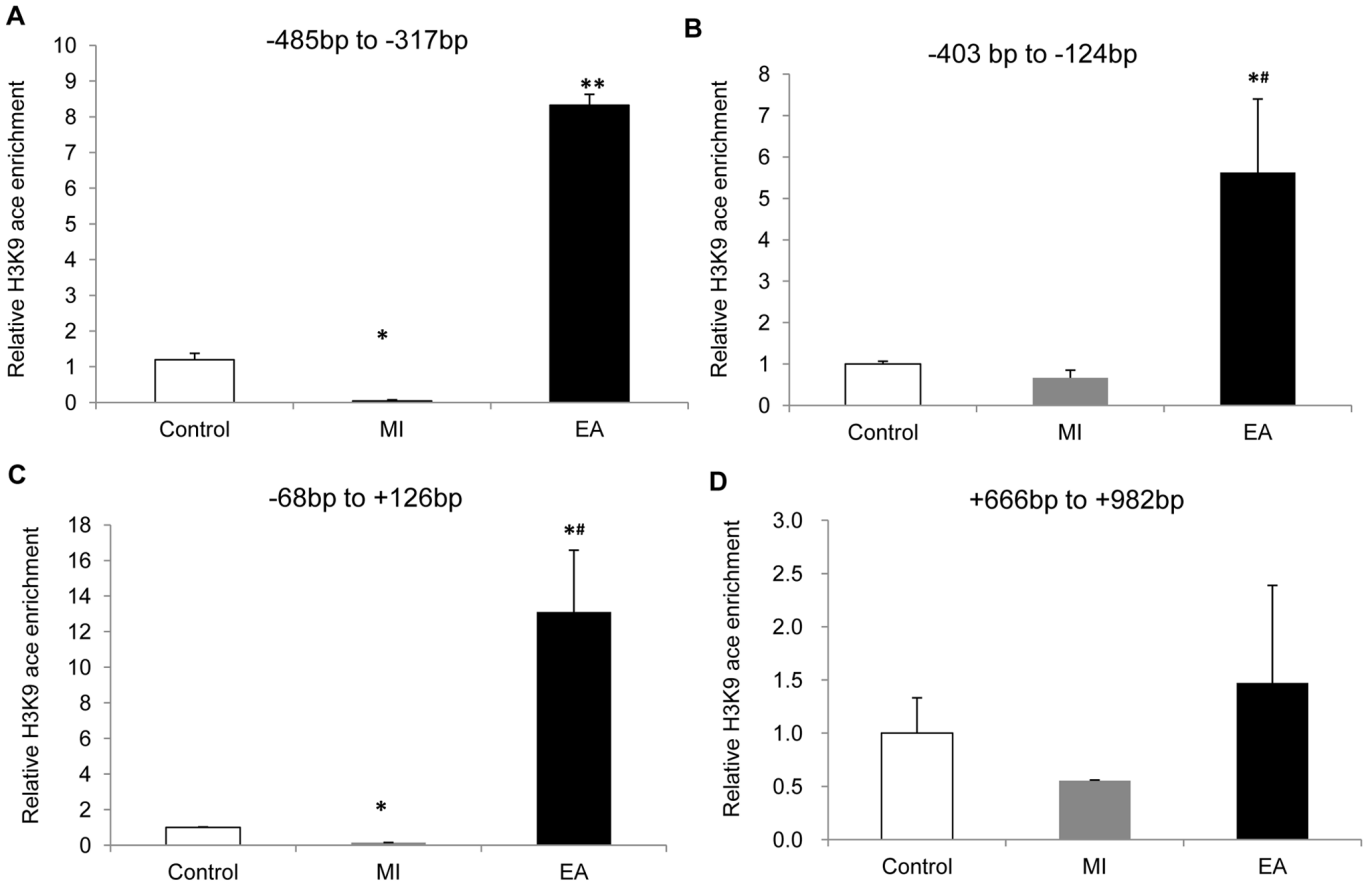
ChIP assay analyses for the enrichment of acetylated H3K9 on the VEGF promoter. For quantitative ChIP analysis, chromatin was extracted from hearts of each group and precipitated with antibodies against H3K9ace. Quantitative PCR was performed to amplify VEGF promoter regions. Results were normalized with respect to input and nonspecific IgG results by using formula [2-(△ Ct) specific antibody/2-(△ Ct) nonspecific IgG], where △Cp is the Cp (immunoprecipitated DNA)- Cp(input) and Cp is the cycle where the threshold is crossed. The bps on the top of each figure shows positions of upstream (+) or downstream (−) of transcription start site on VEGF promoter region (TSS). Data were showed as means ± SD, n = 4, ^*^P<0.05, ^**^P<0.01 vs. Control, ^#^ P<0.01vs MI.

After we validated that VEGF was related to EA-induced angiogenesis, we further explored the mechanism underlying the EA-induced increase of VEGF expression. Western blot results showed that acetylation level of H3K9, an epigenetic marker, was altered according to the expression level of VEGF. The expression of H3K9ace in myocardium decreased in the MI group and the CEA group but increased significantly after EA treatment (*P*<0.01), which corresponded with the changes in VEGF (Fig.6B, 6D). ChIP assay was then applied to study transcriptional and epigenetic regulation during EA-induced angiogenesis. We measured H3K9ace chromatin occupancy at the VEGF promoter region in the infarct myocardial zone (Fig.7). Our results indicated that H3K9ace occupancy at −500 bp to +200 bp region was augmented prominently by EA treatment (P<0.01), suggesting a direct modification of H3K9 acetylation at the VEGF gene. Thus this mechanism might be a major contributor to angiogenesis in ischemic myocardium and the cardioprotective effect of acupuncture.

## Discussion

In light of the theory of Chinese medicine, the use of PC6 in acupuncture is generally advised for the treatment of symptoms of heart and chest diseases, such as palpitation, chest distress, and thoracalgia, nausea, gastralgia, and vomiting. Recently, acupuncture at the PC6 acupoint has been reported to attenuate cardiac injury, such as correcting arrhythmia, reducing apoptosis, and decreasing myocardial enzymes and infarction [9]–[12], which were induced by acute myocardial ischemia. However, the mechanism underlying the protective effects of acupuncture on MI remains unclear.

In order to investigate the effects of acupuncture on MI when the ischemia was in progress instead of stable stage and to explore the epigenetic modification on the MI with or without EA treatment, which should occur before the pathologic changes are stable, we first generated rat MI models by LAD ligation and applied EA intervention to the MI rats. We evaluated myocardial injury by noting ECG's ST segment changes, Q-waves, myocardial enzyme levels, and myocardial histologist. The results suggested that EA treatment decreased ST segment change and Q-wave area, reduced the release of CK, LDH, and CK-MB from the necrosis heart tissue, and alleviated myocardial remodeling. These findings were consistent with previous studies [9]–[12].

After confirming the cardioprotective effect of acupuncture on MI, we explore its possible mechanisms. Genome-wide expression profiles of rats subjected to MI operation and EA treatment were acquired by RNA-seq. According to our RAN-seq data, EA treatment was able to reverse some MI-induced gene expression changes. Pathway analysis indicated that these genes were mainly involved in the calcium signaling pathway, cardiomyopathy related pathways, and the angiogenesis pathway, which was the main interest of this study. Our data indicated a marked increase in VEGF genes expression as a result of EA treatment. Angiogenesis is known as an adaptive response to tissue hypoxia in ischemic myocardial areas, and it is considered a target in treating ischemic diseases, including myocardial ischemia [14], [24]. Acupuncture treatment has also proven to alleviate ischemic symptoms in other types of ischemic diseases via promoting angiogenesis [25], [26]. In the present study, angiogenic responses in ischemic myocardial tissues were examined. Recruitment of cEPCs, as well as proliferation and survival of endothelial cells and vascular smooth muscle cells were necessary for the formation of vascular sprout and lumen. Usually, the high number or density of these cells is indicative of angiogenesis [14]. Our results showed that promoting angiogenesis in ischemic myocardial tissues could be a possible mechanism of the cardioprotective effects of acupuncture in MI cases. Our quantitative analyses for VEGF protein expression levels further confirmed this hypothesis. VEGF-triggered signaling pathways have been well studied. As Cao summarized[27], Ras-Raf-MEK-MAPK is an important cascade for VEGF-induced angiogenesis. Additionally, Akt signaling in endothelial cells also plays critical role in regulating multiple critical steps in angiogenesis, including endothelial cell survival, migration, and capillary-like structure formation[28]. Our genome-wide RNA-seq analysis detected that in accordance with VEGF expression, the expressions of Ras, Akt and several MAPK family members were down-regulated in MI condition and up-regulated by EA treatment (Table 1). Western blot results also showed that VEGF induced Ras-p44/42 MAPK signaling, p38 MAPK signaling, p-JNK signaling and Akt signaling were all activated by EA treatment in MI heart.

Surprisingly and interestingly, the EA treatment on the control rats resulted in reduced expression of VEGF, accompanying with attenuated phospho-p44/42 MAPK and phospho-p38 MAPK expressions. These changes did not induce any pathological phenotypes even these proteins were as low as that in the MI rats. This might be due to a compensatory mechanism by elevating other essential proteins such as Akt and SAPK/JNK, whose signals were much stronger than that in EA group (Figure 6A, B). This compensation did not occur in the ischemic hearts.

To further explore the mechanism underlying the EA-induced increase of VEGF expression, DNA transcriptional and epigenetic regulations during EA-induced angiogenesis were subjected to measurement. Gene expression depends on chromatin structure, which in turn relies on epigenetic modification of histone. In principle, hyperacetylated and decondensed chromatin facilitate gene transcription, whereas nontranscriptionally active regions are often, although not exclusively, hypoacetylated or hypermethylated [29]. Hypoxia induced increase in VEGF expression has been well accepted, and it has recently been found to increase H3K9 acetylation at the VEGF promoters as well [30]. In this study, we found that the alteration of H3K9 acetylation level was consistent with the expression of VEGF. The expression of H3K9ace in ischemic myocardium decreased, but EA treatment could drastically increase its level, along with the retrieval of the VEGF protein. ChIP assay results indicated that H3K9ace occupancy at several regions of VEGF promoter was all increased by MI, and the augmentation of H3K9ace occupancy was prominently magnified by EA treatment. All these data confirmed that acupuncture at PC6 regulates the VEGF expression directly through H3K9 acetylation. Similarly, a decreased level of acetylation of H3K9 was observed in EA treated control rats (Figure 6C, D), suggesting a strong candidate for mediating the decrease of VEGF, phospho-p44/42 MAPK and phospho-p38 MAPK expressions. It indicated that pre-treatment of electroacupuncture might be a stimulation to initiate a protective effect on ischemic hearts, similar as an ischemic preconditioning [31]–[33]. A further study for this hypothesis might be of significance.

## Conclusions

**Figure 8:**
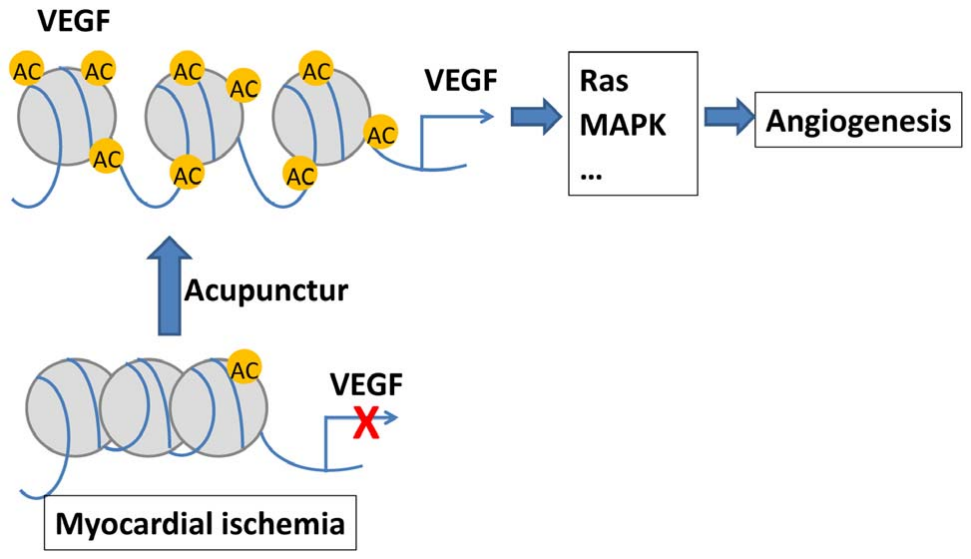
Scheme of a possible mechanism for EA on myocardial ischemia through VEGF signaling.

In summary, our data indicate that acupuncture exerts a cardioprotective effect in MI rats by promoting angiogenesis, which is mediated through H3K9 acetylation modification directly at the VEGF promoter (Fig.8). We also present evidence that histone modification plays a role in the cardioprotective effects of EA upon MI injury, and H3K9 acetylation can be a therapeutic target for EA or cardioprotective medicines following myocardial ischemia, although additional molecular experiments may be needed to verify its therapeutic potentials. In addition, our study provides, for the first time, informative genome-wide profiles of gene expressions in MI injury and electroacupuncture treatment that can be used in future studies. Further ChIP-seq analyses will be employed to investigate genome-wide H3K9ace modification and other epigenetic marks that could regulate gene expressions in the EA treatment of MI cases.

## Supporting Information

Figure S1 **Blood pressure change after ligation of LAD.** During the operation, rats blood pressure were monitored by the carotid artery intubation, data were expressed as means ± SD (n = 10), ^**^ *P*<0.01 *vs.* before operation. A. Carotid artery diastolic; B. systolic blood pressure.
(TIF)

Figure S2 **Survival rate of rats in each group.** The survival number of each group was recorded very day after operation, and the survival rate of each group was calculated with the formula: (survival rat number/the total rat number) ×100%. Data were expressed as means ± SD, n = 10∼32, significantly compared *P*<0.001, EA group vs. MI group.
(TIF)

Figure S3 **Representative Hematoxylin and Eosin (H&E) staining results of each group.** Cardiac tissues were collected at the end of EA treatment for 7 days, and prepared for H&E staining. The sections from the apex (a to d, 40 magnification), mid-left ventricle (e to h, 40 magnification), and the ventricular wall (i to p, 200 magnification) were shown.
(TIF)

Figure S4 **Representative Masson's trichrome staining results of each group.** Cardiac tissues were collected at the end of EA treatment for 7 days, and prepared for Masson's trichrome staining. The sections showed the results by different amplifications (a to d, 40 magnification, e to h, 100 magnification,i to l, 200 magnification, m to p, 400 magnification).
(TIF)

Figure S5 **Schematic diagram of rats under the EA treatment on PC6.**
(TIF)
