# Supplementary figures and images for: Acupuncture Promotes Angiogenesis after Myocardial Ischemia through H3K9 Acetylation Regulation at VEGF Gene
Source: PLoS One. 2014 Apr 10;9(4):e94604. doi: 10.1371/journal.pone.0094604 (PMC3983235; doi:10.1371/journal.pone.0094604)

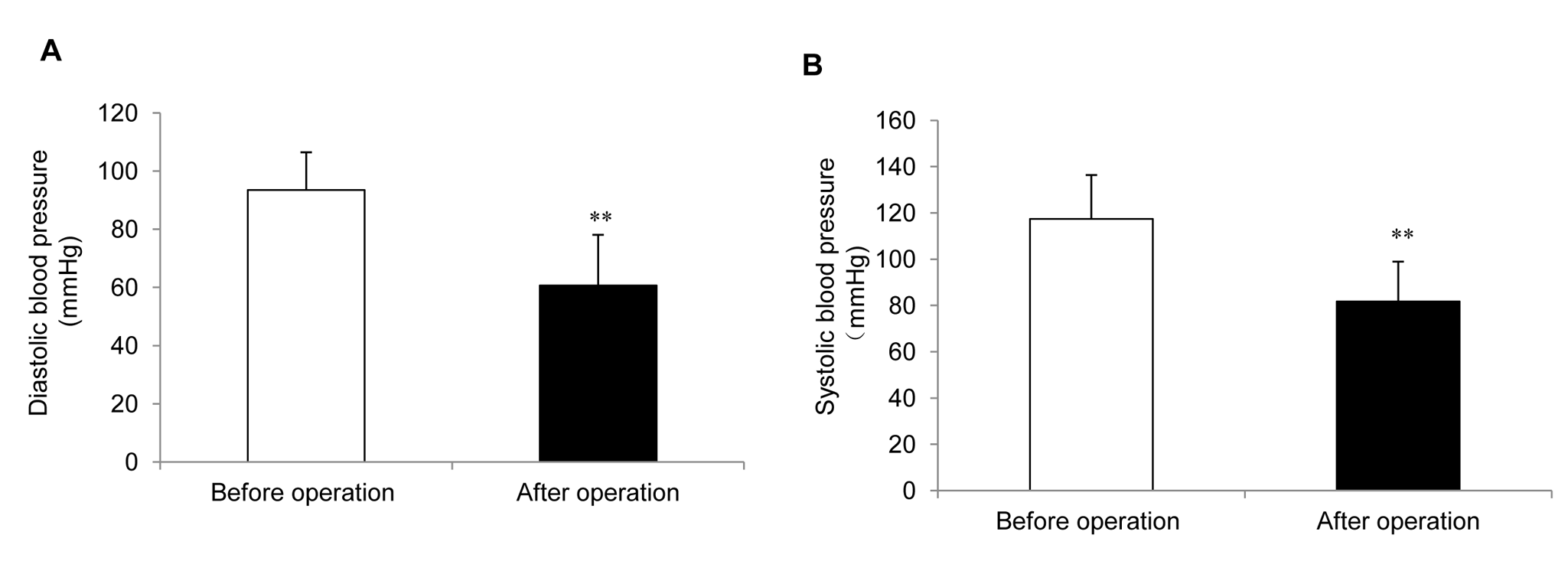

Supplement: Figure S1 — Blood pressure change after ligation of LAD. During the operation, rats blood pressure were monitored by the carotid artery intubation, data were expressed as means ± SD (n = 10), ** P<0.01 vs. before operation. A. Carotid artery diastolic; B. systolic blood pressure. (TIF) [file pone.0094604.s001.tif]

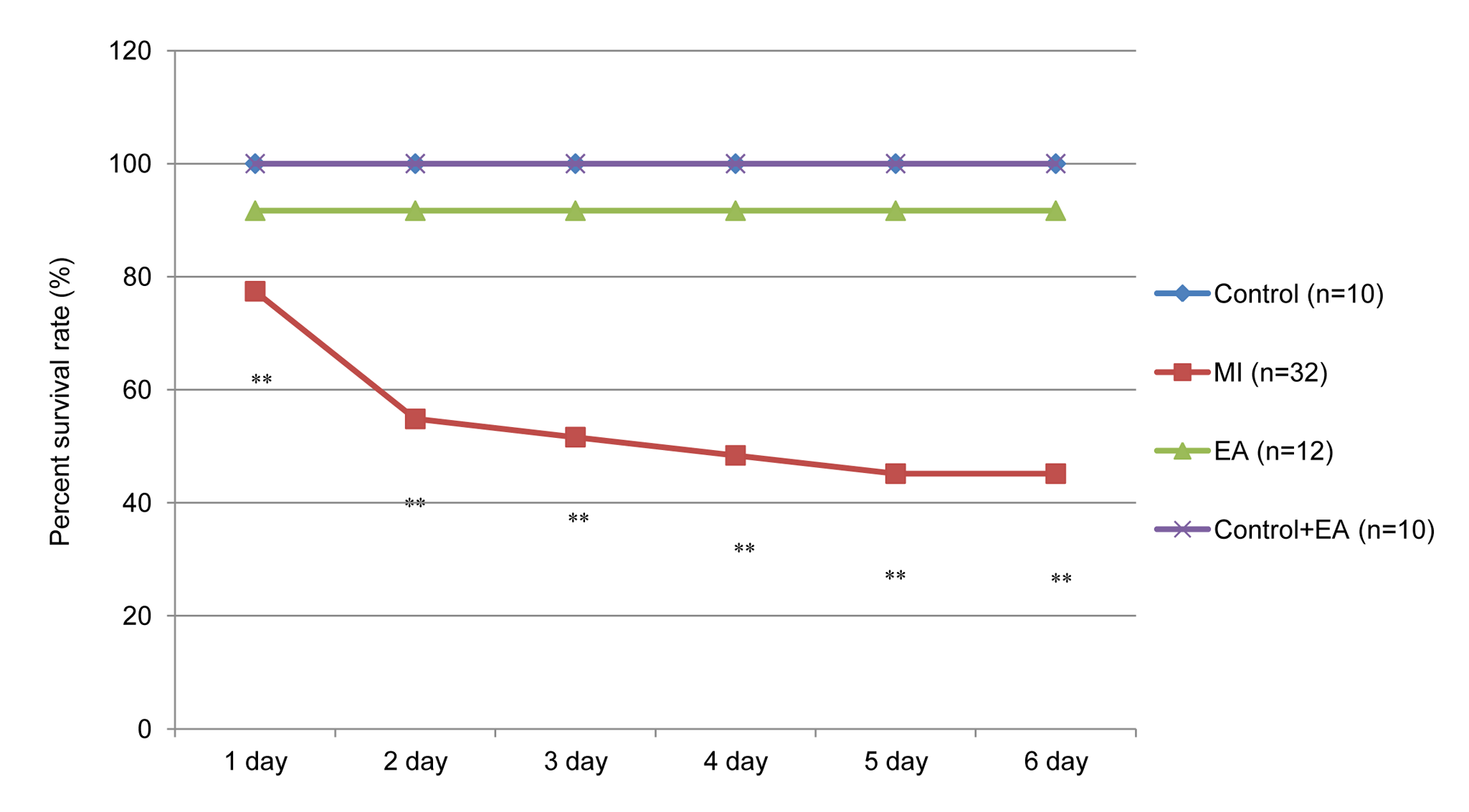

Supplement: Figure S2 — Survival rate of rats in each group. The survival number of each group was recorded very day after operation, and the survival rate of each group was calculated with the formula: (survival rat number/the total rat number) ×100%. Data were expressed as means ± SD, n = 10∼32, significantly compared P<0.001, EA group vs. MI group. (TIF) [file pone.0094604.s002.tif]

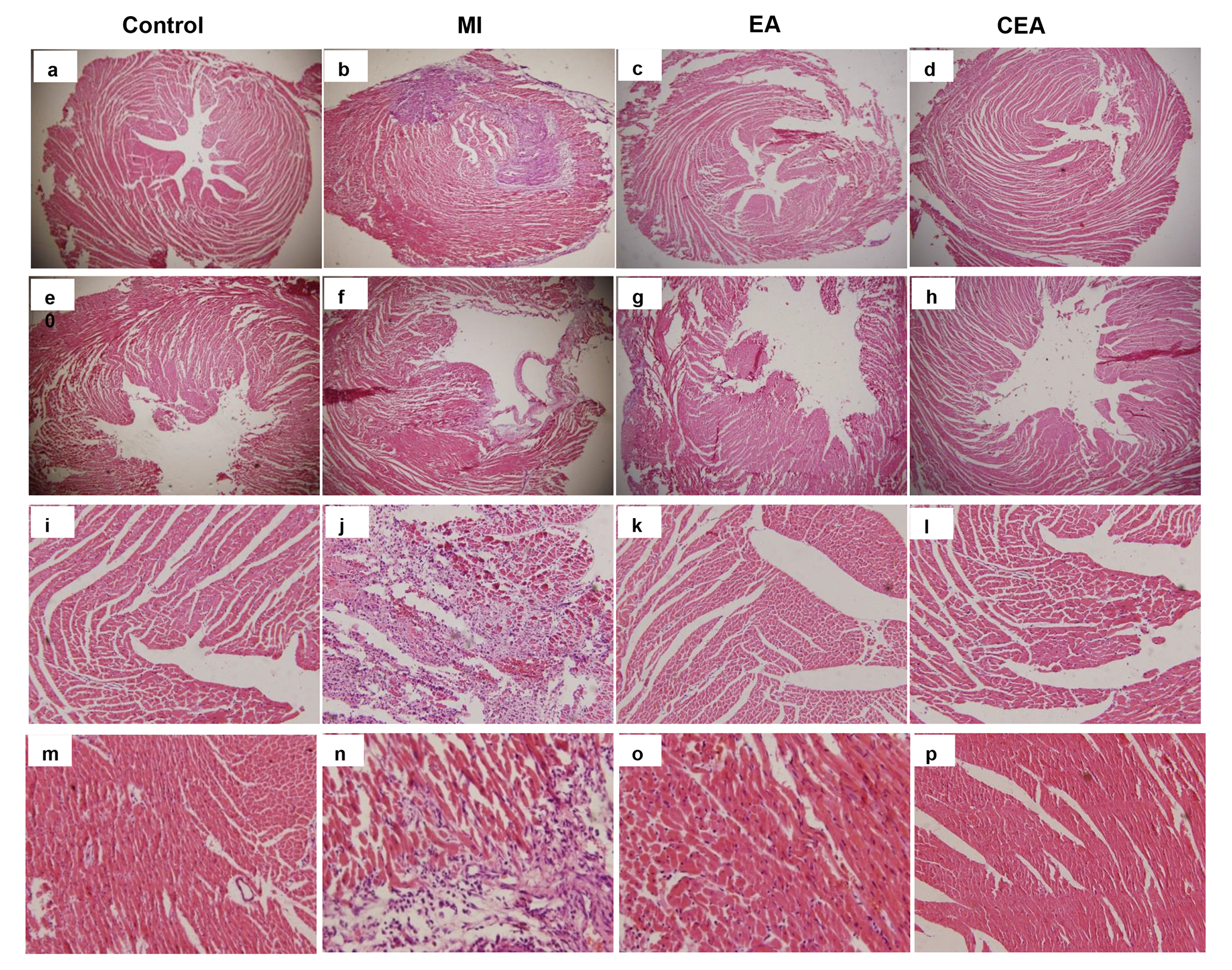

Supplement: Figure S3 — Representative Hematoxylin and Eosin (H&E) staining results of each group. Cardiac tissues were collected at the end of EA treatment for 7 days, and prepared for H&E staining. The sections from the apex (a to d, 40 magnification), mid-left ventricle (e to h, 40 magnification), and the ventricular wall (i to p, 200 magnification) were shown. (TIF) [file pone.0094604.s003.tif]

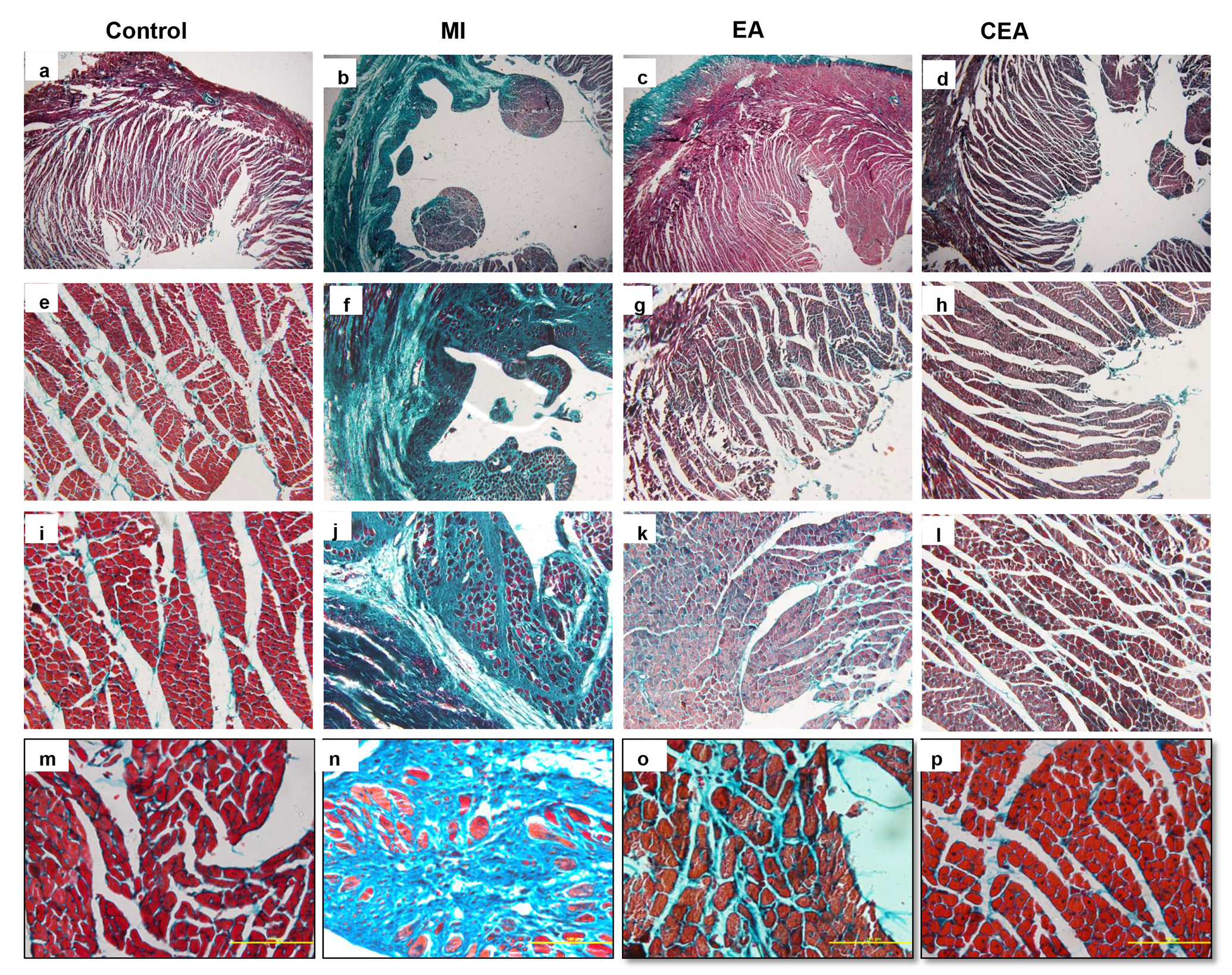

Supplement: Figure S4 — Representative Masson's trichrome staining results of each group. Cardiac tissues were collected at the end of EA treatment for 7 days, and prepared for Masson's trichrome staining. The sections showed the results by different amplifications (a to d, 40 magnification, e to h, 100 magnification,i to l, 200 magnification, m to p, 400 magnification). (TIF) [file pone.0094604.s004.tif]

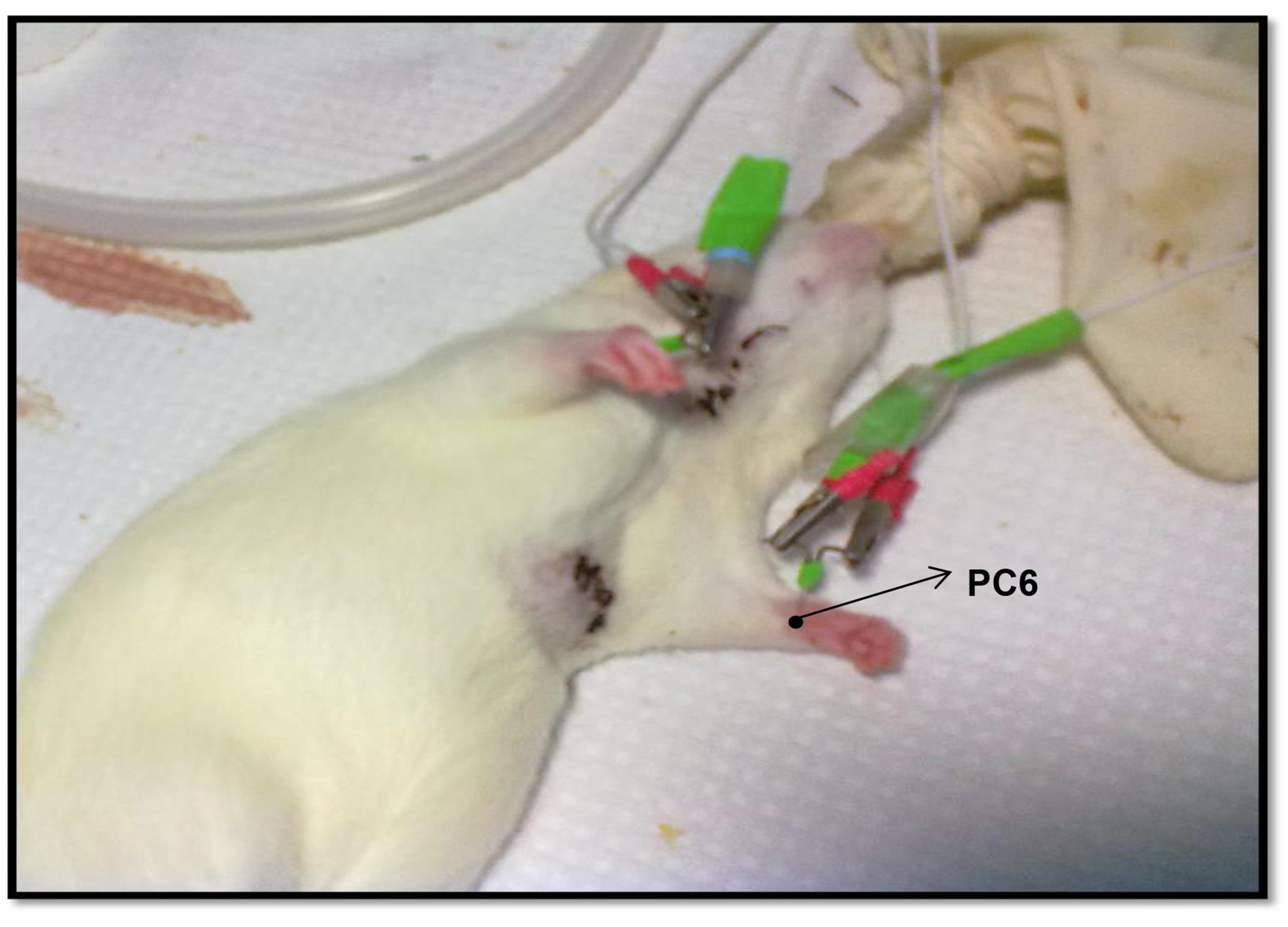

Supplement: Figure S5 — Schematic diagram of rats under the EA treatment on PC6. (TIF) [file pone.0094604.s005.tif]
